# A High-Throughput, Multi-Cell Phenotype Assay for the Identification of Novel Inhibitors of Chemotaxis/Migration

**DOI:** 10.1038/srep22273

**Published:** 2016-03-09

**Authors:** Xin-Hua Liao, Netra Pal Meena, Noel Southall, Lunhua Liu, Manju Swaroop, Arina Li Zhang, Jan Jian Xiang, Carole A. Parent, Wei Zheng, Alan R. Kimmel

**Affiliations:** 1 Institute for Translational Medicine, School of Basic Medical Sciences, Fujian Medical University, Fuzhou, Fujian 350108, China; 2 Laboratory of Cellular and Developmental Biology, National Institute of Diabetes and Digestive and Kidney Diseases, The National Institutes of Health, Bethesda, MD 20892, USA; 3 Therapeutics for Rare and Neglected Diseases, National Center for Advancing Translational Sciences, The National Institutes of Health, Bethesda, MD 20892, USA; 4 Laboratory of Cellular and Molecular Biology, National Cancer Institute, The National Institutes of Health, Bethesda, MD 20892, USA

## Abstract

Chemotaxis and cell migration are fundamental, universal eukaryotic processes essential for biological functions such as embryogenesis, immunity, cell renewal, and wound healing, as well as for pathogenesis of many diseases including cancer metastasis and chronic inflammation. To identify novel chemotaxis inhibitors as probes for mechanistic studies and leads for development of new therapeutics, we developed a unique, unbiased phenotypic chemotaxis-dependent *Dictyostelium* aggregation assay for high-throughput screening using rapid, laser-scanning cytometry. Under defined conditions, individual *Dictyostelium* secrete chemoattractants, migrate, and aggregate. Chemotaxis is quantified by laser-scanning cytometry with a GFP marker expressed only in cells after chemotaxis/multi-cell aggregation. We applied the assay to screen 1,280 known compounds in a 1536-well plate format and identified two chemotaxis inhibitors. The chemotaxis inhibitory activities of both compounds were confirmed in both *Dictyostelium* and in human neutrophils in a directed EZ-TAXIscan chemotaxis assay. The compounds were also shown to inhibit migration of two human cancer cell lines in monolayer scratch assays. This test screen demonstrated that the miniaturized assay is extremely suited for high-throughput screening of very large libraries of small molecules to identify novel classes of chemotaxis/migratory inhibitors for drug development and research tools for targeting chemotactic pathways universal to humans and other systems.

Chemotaxis is a fundamental process whereby cells sense and migrate in chemoattractant gradients. Chemotaxis and cell migration play pivotal roles in embryogenesis, inflammation, wound healing, and renewal of skin and intestinal cells. They also mediate development of chronic inflammatory diseases, such as asthma, chronic obstructive pulmonary disease, rheumatoid arthritis, and atherosclerosis, as well as, cancer, angiogenesis, and metastasis^1^. Chemotaxis is regulated by G protein coupled receptors (GPCRs) and heterotrimeric G proteins that transduce chemotactic signals to the cytoskeleton to dynamically polarize migratory cells. Such biased polarizations may help re-localize the intracellular machinery for basal cell motility toward directed movement^2^. The molecular mechanisms that regulate chemotaxis/migration under different biological and pathological conditions are complex, and discovering novel small molecule probes of these pathways is important to analyze mechanistic functions and to develop new therapeutics^1^,^3^,^4^,^5^,^6^,^7^,^8^. Pathways involving ligand sensing, signal transduction, and basal cell mobility may all be targets for inhibition. Previous *in vitro* drug screens for anti-inflammatory and anti-metastasis potential have often focused on chemokine receptors, adhesion molecules, and limited downstream pathways^4^,^5^,^6^,^7^,^8^. However, the compounds identified from molecular target-based screens generally have poor activity *in vivo* and only few of them have been advanced to clinical trials.

Several cell-based migration assays are being optimized for more high-throughput image screening^9^,^10^,^11^,^12^,^13^,^14^,^15^,^16^,^17^,^18^,^19^,^20^, but they are not yet compatible to screen 1000s of compounds across a broad range of concentrations. Microfluidic devices are easily automated and show promise, but have been difficult to scale beyond 96 format arrays^11^,^12^,^18^,^21^. Magnetically labeled cell groups can be cultured in 3D, disrupted, and allowed to coalesce in a ring closure assay that assesses cell motility. The assay can be performed in 96-well plates, but requires magnetic and mechanical manipulations^14^. Boyden chambers have been re-configured to 96-well formats, but whole single-plate image analysis is limited and cell migration efficiency can be restricted to ~20%^9^,^10^.

Here, we report a simple, phenotypic, fluorescent chemotaxis-dependent aggregation assay in a 1536-well plate format that utilizes the unique chemotactic properties of *Dictyostelium*. Chemotaxis has been comprehensively studied in *Dictyostelium discoideum*, a model organism for eukaryote development, cell differentiation, and chemotaxis^1^,^22^,^23^,^24^,^25^,^26^, that shares similar chemotactic mechanisms with mammalian migratory cells^1^,^26^. *Dictyostelium* has also proven to be a highly sensitive system to evaluate the effects of various compounds on chemotaxis^27^,^28^,^29^,^30^.

*Dictyostelium* has a unique life cycle involving unicellular growth and multicellular development. *Dictyostelium* cells grow individually under nutrient abundant conditions, but upon starvation, they enter a developmental program and secrete the chemoattractant cAMP, which directs cell-to-cell chemotactic migration, formation of tight multi-cell aggregates, and multicellular development, with the terminal differentiation of distinct cell classes^1^,^22^,^23^,^24^,^25^. A GFP reporter, which is only expressed upon chemotactic-mediated aggregation^31^, is used to assess chemotaxis-dependent aggregation (Fig. 1A and Supplementary Movie 1). The assay was miniaturized and automated to a 1536-well plate format, where GFP fluorescence can be rapidly quantified (Fig. 1). A viability counter screen was also developed and incorporated, to eliminate cytotoxic false positives.

While the described *Dictyostelium* chemotaxis-dependent aggregation assay system offers unique advantage for HTS, we recognize that the readout is indirect. Still, compounds that affect development independently of chemotaxis, signal-response, and motility are easily eliminated in directed screens. Further, we demonstrate successful proof of application, using mammalian cells as readouts.

In a test screen of 1280 small molecules, at 7 (*i*.*e*. >4-log) concentrations each, using the described *Dictyostelium* chemotaxis-dependent aggregation assay system, we identified two compounds that inhibit chemotaxis in mammalian cells. The inhibitory activity of both compounds was independently confirmed using an EZ-TAXIScan chemotaxis assay^13^,^32^, applied to *Dictyostelium* and human neutrophils, and a scratch migration assay^33^, applied to human migratory cancer cells. The *Dictyostelium* aggregation assay is a first assay suitable for HTS of chemotaxis/migration inhibitors in large libraries of small molecules, and we have described a novel technological approach to identify lead compounds in chemotaxis for mechanistic studies and new therapeutics.

## Results

### Principles of Assay and Signal Detection

**Figure 1:**
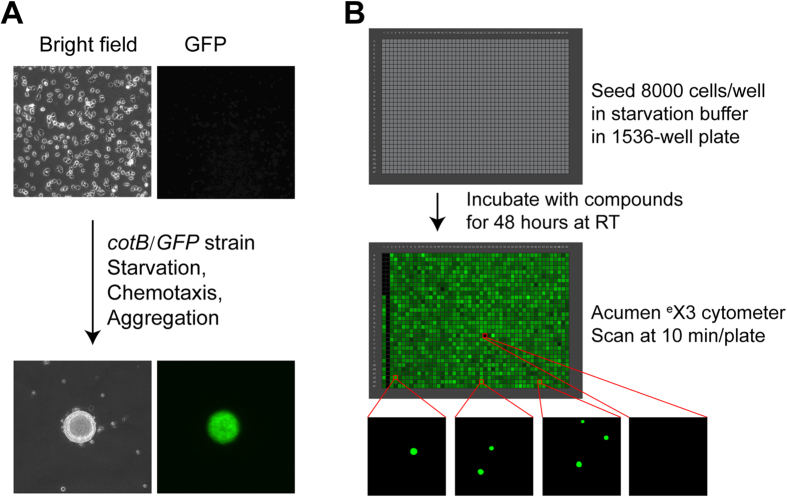
Imaging GFP reporter-based *Dictyostelium* chemotaxis-dependent aggregation assay for chemotaxis inhibitor screening. (**A**) Principle of the screening assay. **Top:** Single cells before aggregation (bright field microscopy) do not express the *cotB*/*GFP* reporter. **Bottom:** Under starvation conditions, *Dictyostelium* secrete the chemoattractant cAMP, chemotax, and form multicellular aggregates, which express GFP after 24 hr. (**B**) Protocol for compound screening. **Top:** 1536-well Acumen ^e^X3 GFP image at the start of the experiment. In general, wells contained 8,000 *Dictyostelium* cells. The left-most vertical row of cells has decreasing concentrations of latrunculin A (see Fig. 3A). The second (from left) vertical row of cells has the highest concentration of latrunculin A in all wells, as a negative signal (*i*.*e*. a full inhibitory) control. The third and fourth vertical rows of cells have 0.38% DMSO, as positive signal controls (*i*.*e*. without any inhibition). All other wells contain different small molecule compounds. **Middle:** An entire, 1536-well Acumen ^e^X3 GFP image after 48 hr incubation. **Bottom:** Selected Acumen ^e^X3 GFP images of individual wells. GFP positive, usually form 1–3 multicellular *Dictyostelium* aggregates within each well of a 1536-well plate format, while no aggregates form when chemotaxis is inhibited. Criteria are set to quantify GFP signals in objects >30 μM diameter; non-aggregated cells have a zero GFP signal. Each image is an entire well, 1.53 mm × 1.53 mm.

Upon nutrient depletion, *Dictyostelium* initiate a multicellular developmental program involving secretion of the chemoattractant cAMP that directs chemotaxis and tight multi-cell aggregation. This morphological transition from unicellular to multicellular state is easily observed (Fig. 1A). Chemotaxis/aggregation is quantified using *Dictyostelium* carrying a stable GFP reporter (*cotB*/*GFP*)^31^ that is only expressed in multicellular aggregates (Fig. 1A). We miniaturized and automated the assay (Supplementary Table 1) to a 1536-well plate format that allows the formation of only 1–3 (GFP-positive) aggregates per well in a limited surface area (2.3 mm^2^ and 6 μl media per well; Fig. 1B).

We evaluated three different instruments to rapidly quantify GFP signals following chemotaxis/aggregation in 1536-well plates. The ViewLux CCD imager based fluorescence plate reader was not sufficiently sensitive to detect the GFP signals. The 1 to 3 GFP-aggregates per well were frequently localized outside the detection field of the EnVision photomultiplier tube based fluorescence plate reader. In contrast, GFP-aggregation fluorescent signals were readily detected by the Acumen ^e^X3 laser scanning based plate cytometer, with full data collection of an entire 1536-well plate within ~10 min.

Scanning threshold was set to capture signals from objects greater than 30 μm in diameter (>3 cell equivalents) and record numbers of aggregates, total fluorescence intensity, and peak fluorescence intensity of aggregates within each well (Fig. 1B). The GFP peak intensity per well proved to be the most consistent, on well-to-well and plate-to-plate bases, and was chosen as the primary readout.

In addition to high throughput and quick data acquisition, the phenotypic screening approach provides further advantages. Since GFP is only expressed in multicellular aggregates (not individual cells, see Fig. 1A), fluorescent signals are highly concentrated to the limited multi-cellular foci (aggregates) within each well (Fig. 1 and Supplementary Movie 1). GFP-positive aggregates are readily distinguished from non-chemotaxing, GFP-negative individual cells. Because there is no background GFP signal below the 30 μm diameter detection threshold, compounds inhibiting chemotaxis would ablate strong positive GFP signals.

### Assay Automation, Miniaturization, and Optimization

#### Time course

**Figure 2:**
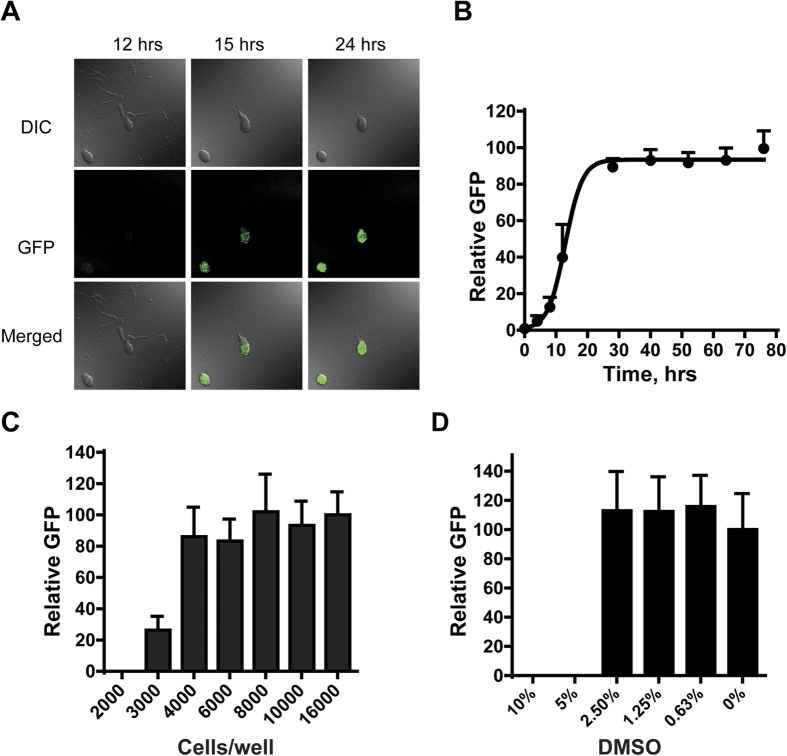
Optimization of *Dictyostelium* chemotaxis-dependent aggregation assays. (**A**) Time course of reporter GFP expression during *Dictyostelium* chemotaxis and aggregation. Cells were incubated in starvation buffer for various times and visualized by differential interference contrast (DIC) and fluorescent confocal (GFP) microscopy (see Supplementary Movie 1). (**B**) For measuring time course of GFP expression in 1536-well plate format, cells were seeded into plates and assayed at times indicated for GFP signal intensity (with standard deviations) with the Acumen ^e^X3 laser scanning cytometer. The data are representative of two independent experiments. (**C**) For cell density optimization, cells were deposited in 1536-well plates at different densities and GFP intensity (with standard deviations) measured after 48 hr. (**D**) For evaluation of DMSO effects, 8,000 cells per well were incubated with different concentrations of DMSO and GFP intensity (with standard deviations) was measured after 48 hr.

To evaluate timing, we seeded *Dictyostelium* in starvation/differentiation buffer in 1536-well plates, at 6 μl/well, and incubated at ~20 °C (Supplementary Table 1). Chemoattractant (cAMP) signaling was initiated endogenously after 8 hr and resulted in cells chemotaxing and aligning in a head-to-tail mode (*i*.*e*. cell-to-cell streaming; Fig. 2A and Supplementary Movie 1)^34^. GFP expression was observed in early aggregated cells and fluorescence intensity increased with incubation times (Fig. 2A,B and Supplementary Movie 1). The maximal GFP expression in the 1536-well format was observed after ~30 hr incubation and remained constant for an additional 2 days (Fig. 2B). We chose a 48-hr incubation time to ensure that all aggregates of entire plates attained peak GFP signals.

#### Cell density

To minimize well-to-well variation and ensure reproducibility, we optimized cell density for chemotaxis-dependent aggregation in small surface areas of 1536-plate wells. Cells at low density (<2,000 cells/well) failed to aggregate or express GFP (Fig. 2C). The peak GFP signal intensity increased from 3,000 to 8,000 cells/well, and plateaued (Fig. 2C). Our data also indicated that at cell densities >12,000 cells/well, aggregation and GFP expression could occur, even with poor chemotaxis. Thus, 8,000 cells/well (~3.5 × 10^5 ^cells/cm^2^) in 6 μl buffer was chosen as optimal for the 1536-well plate format (Fig. 2C and Supplementary Table 1). This cell density was proportionally similar to that previously developed for 12-well plates (~1.5 × 10^5 ^cells/cm^2^)^35^. Approximately, 1–3 aggregates with variable sizes form in each well of a 1536-well plate under optimal conditions (Fig. 1B).

#### Temperature sensitivity

Multi-cell aggregate formation was robust and reproducible at 20–23.5 °C. Our results were consistent with the reported optimum growth temperature for *Dictyostelium* at ~22 °C. *Dictyostelium* undergo heat-shock at 28 °C^36^; chemotaxis is significantly delayed at <15 °C.

#### Assay verification

Since DMSO was used as the universal solvent for all compounds, we examined DMSO effects on aggregation in 1536-well plate formats. Neither aggregation nor GFP expression was affected at final DMSO concentrations <2.5% (Fig. 2D). The DMSO concentration in our compound screens is 0.38% (Supplementary Table 1).

Since the GFP-expressing multicellular aggregates formed within each well have different sizes and numbers, the total fluorescence intensity signals can vary [coefficient of variation (CV) = 27%)] from well-to-well and plate-to-plate, with a Z’ factor of 0.47. Importantly, the parameters of the laser scanning plate cytometer are set to only quantify GFP fluorescence presented in multicellular aggregates. The design of the assay results in a “zero” signal when aggregation is completely inhibited (see Fig. 1) and, consequently, in non-conventional signal-to-basal ratio calculations. This high throughput assay is extremely robust, sensitive, and reliable for the identification of novel chemotaxis inhibitors within small molecule libraries.

**Figure 3:**
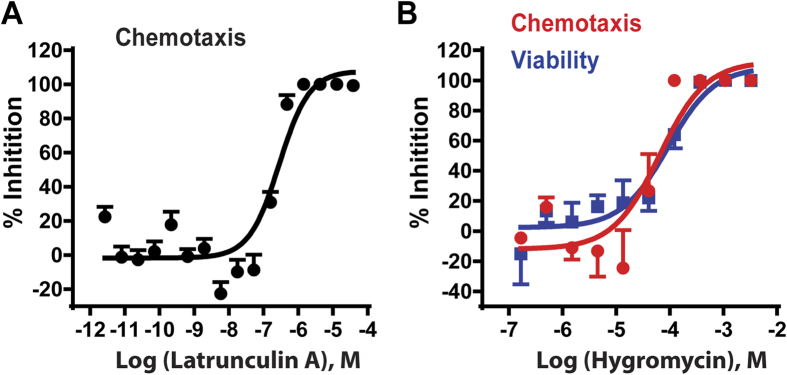
Validation of the *Dictyostelium* chemotaxis-dependent aggregation assay for inhibitor screens. (**A**) Effects of latrunculin A on chemotaxis-dependent aggregation (GFP expression). 8,000 cells per well were incubated with different concentrations of the chemotaxis inhibitor latrunculin A in the 1536-well plate. GFP intensity (with standard deviations) was measured after 48 hr. (**B**) ATP content assay as a counter screen for cytotoxicity. Parallel wells with different concentrations of the cytotoxic compound hygromycin B were assayed for viability (500 cells/well) after 3 days and chemotaxis-dependent aggregation (8,000 cells/well) after 2 days. Inhibition of cell viability was assayed by loss of cellular ATP and inhibition of aggregation was assayed by loss relative GFP intensity. The viability and aggregation inhibition curves (with standard deviations) superimpose.

Latrunculin A is a natural product, which binds actin monomers at 1:1 with a Kd of 200 nM *in vitro*^37^. Inhibition of actin polymerization by latrunculin A disrupts actin filament formation, cytoskeletal organization, cell migration, and chemotaxis^15^,^38^. We evaluated inhibitory activity of latrunculin A on chemotaxis-dependent aggregation in the 1536-well format. Latrunculin A inhibited chemotaxis-dependent aggregation with an IC_50_ of 282 nM (95% confidence interval, 193–412 nM; Fig. 3A), similar to that of mammalian cells. Results demonstrated that the *Dictyostelium* assay is an effective alternate approach for compound screening to identify chemotaxis inhibitors. While latrunculin A may cause lethality of oncogenic mammalian cells, it is not toxic to *Dictyostelium* in our assay. When *Dictyostelium* are seeded in starvation buffer, growth is arrested and non-dividing cells survive without actin-dependent processes for external nutrient capture, cell division, or motility; cellular energy, etc. derive from autophagy. When we simultaneously monitored dose response effects of latrunculin A on aggregation and cell viability, complete inhibition of aggregation is confirmed at 1 μM, whereas cells remained viable to 93+/−2.3%, as assayed by vital dye staining^39^. Latrunculin A is more cytotoxic at 10 μM (~30% viability).

We do not observe partial inhibition of GFP expression by lactrunculin A across a broad concentration range (Fig. 3A), as might have been anticipated; in addition, smaller aggregates are not a characteristic of inhibition. The non-linearity of the detected GFP signal response is intrinsic to this unique phenotypic assay and also provides significant advantage. First, cell-cell communication (signal-relay) between individual *Dictyostelium* is essential to chemotaxis-dependent aggregation and both signal-relay and chemotaxis are regulated by many of the same intracellular pathways^22^,^23^,^24^,^26^,^40^. Thus, inhibitory effects can be amplified if they impact both chemotaxis and signal-relay, as does latrunculin A^34^,^41^.

In addition, since the assay proceeds for 48 hrs, we lessen minimal inhibitory effects on chemotaxis-dependent aggregation and GFP expression. Finally, GFP is only detected in objects >30 μM, which further emphasizes positive/negative contrasts. We suggest that these criteria polarize effects toward either non-inhibitory or highly inhibitory signals (Figs 1B and 3A). Since we test compounds across a wide concentration range, these data enhance discrimination of true inhibitory molecules from false positives.

#### Counter screen to eliminate toxic compounds

Since compounds that cause cell lethality will appear as inhibitory positives in our assay, we adapted a cell-based ATP content assay to evaluate compound cytotoxicity to eliminate those false positives from a primary screen^42^. Since *Dictyostelium* cells within aggregates were not easily lysed with the assay-kit buffer, we established an ATP content assay (Supplementary Table 2) using 500 cells/well in which aggregation was not formed due to non-permissive numbers of cells (Fig. 2C). Hygromycin B was used as a positive control for cytotoxicity that exhibited similar IC_50_ values of 92.8 μM and 64.5 μM in the ATP content and aggregation assays, respectively (Fig. 3B). Our results indicate that 3-day incubation is optimal for measurement of compound cytotoxicity using the ATP content assay. Hygromycin B was then used as a parallel cytotoxic control in all experiments. The signal-to-noise ratio was 22-fold, CV was 12%, and Z’ factor was 0.6 in this *Dictyostelium* cytotoxicity assay.

### LOPAC collection screening

**Figure 4:**
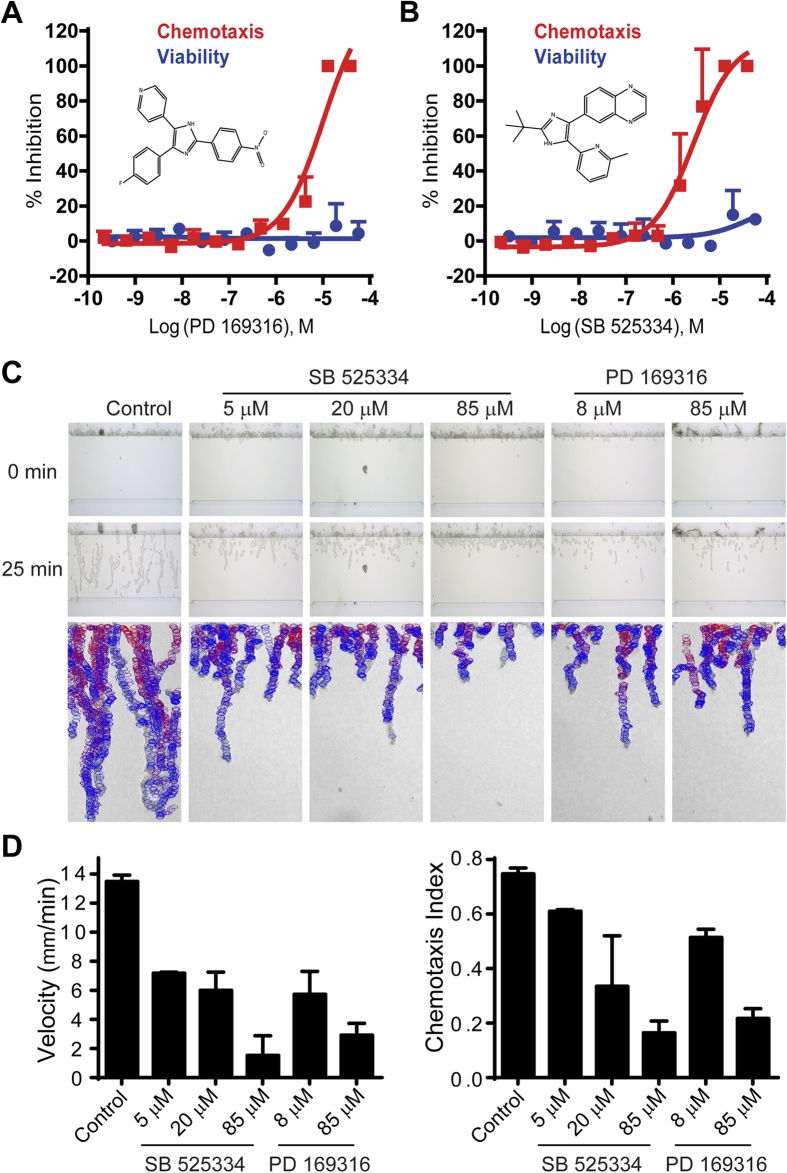
Identification and confirmation of *Dictyostelium* chemotaxis inhibitors PD 169316 and SB 525334 from LOPAC screening. (**A,B**) Concentration response of PD 169316 (**A**) and SB 525334 (**B**) with strong inhibitory effects on chemotaxis-dependent aggregation (GFP), without obvious effect on cell viability (ATP). The experiments were performed in triplicate. Error bars represent standard deviations. Structures of PD 169316 and SB 525334 are embedded in the graphs. (**C**) Validation of inhibitory effects of compounds PD 169316 and SB 525334 on *Dictyostelium* chemotaxis in EZ-TAXIScan assays. Developed *Dictyostelium* were treated with various doses of PD 169316 and SB 525334 and assayed for chemotaxis within a cAMP gradient by EZ-TAXIScan. **Top and middle panels:** the images show the positions of cells at 0 and 25 min of chemotaxis; at 25 min, control cells approach the end of the gradient (see Figure S2A). **Bottom panels:** the images show migratory paths of individual *Dictyostelium* during an entire chemotaxis assay. Cells are depicted in migratory tracts as circles, progressing from red to blue during increasing times of the assay. For clarity, only cells that moved in ten consecutive frames are shown. Non-moving (*e*.*g*. stationary) features are ignored by the algorithm, which compares objects among multiple images. Data are representative of three independent experiments. (See Supplementary Movie 2). (**D**) Chemotaxis parameters were calculated using data obtained from (**C**). The chemotaxis velocity was computed as the total chemotaxis path length divided by time. The chemotaxis index was computed as the ratio of net path length toward cAMP compared to the total path length.

The LOPAC (Library of Pharmacologically Active Compounds) collection contains 1280 compounds commonly used to test and validate screen assays. We performed a primary *Dictyostelium* chemotaxis-dependent aggregation assay with all 1280 LOPAC compounds in serial dilution (from ~2 nM to ~50 μM) in parallel with an ATP content cytotoxicity assay. Based on calculation of IC_50_, three compounds fully inhibited chemotaxis/aggregation at ≤1.5 μM, but all three were also cytotoxic; at ≤7.7 μM, 22 compounds were inhibitory, with 20 also cytotoxic (see Figure S1A,B). The inhibitory effects on aggregate formation of the two non-toxic compounds PD 169316 and SB 525334 (Fig. 4A,B) were confirmed by microscopic examination; PD 169316 and SB 525334 did not simply suppress GFP expression, but also inhibited chemotaxis-dependent aggregation.

Finally, while 123 other compounds were inhibitory at only the single highest dose tested, 38.3 μM, 111 of these compounds were also cytotoxic; we did not pursue the remaining 12 compounds, rather focusing on the more active compounds PD 169316 and SB 525334.

PD 169316 and SB 525334 were individually re-assayed for dose-response inhibition of aggregation and cytotoxicity over a wider concentration range (Fig. 4A,B). PD 169316 (Fig. 4A) inhibited *Dictyostelium* aggregation with an IC_50_ of 10 μM (95% confidence interval, 5.1–19.7 μM) with no detectable cytotoxicity. SB 525334 (Fig. 4B) also strongly inhibited aggregation (IC_50_ 2.8 μM, 95% confidence interval, 1.5–5.1 μM). The LOPAC test screen demonstrated the suitability of the *Dictyostelium* chemotaxis-dependent aggregation assay in 1536-well plate formats for HTS to identify potential inhibitors of chemotaxis.

### PD 169316 and SB 525334 inhibit chemotaxis of both *Dictyostelium* and human neutrophils

**Figure 5:**
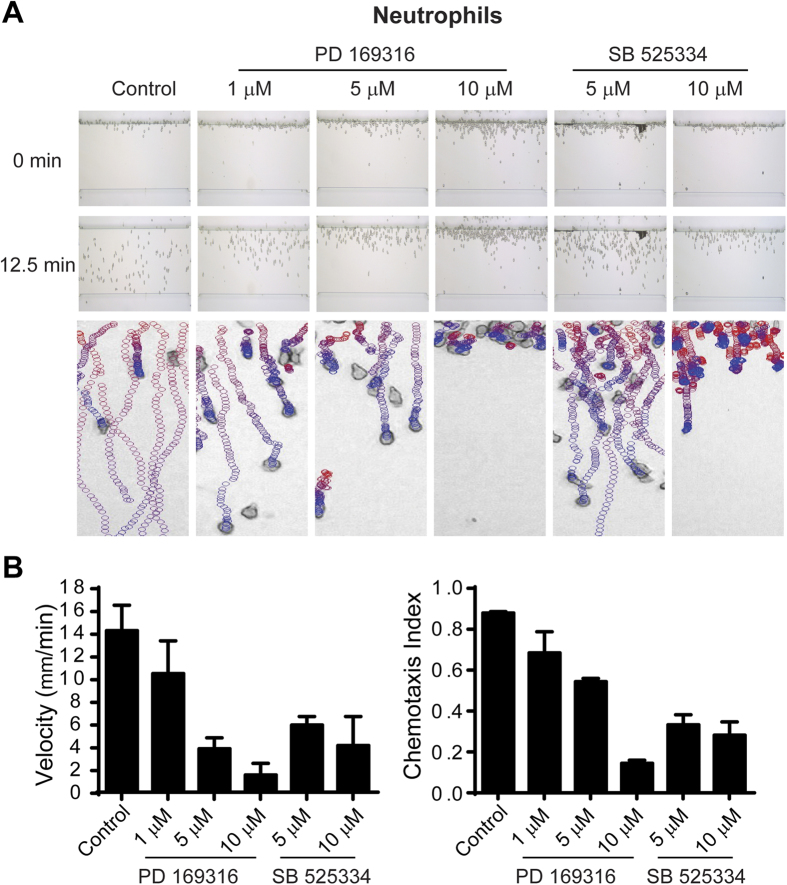
Validation of inhibitory effects of compounds PD 169316 and SB 525334 on human neutrophil chemotaxis in EZ-TAXIScan assays. (**A**) Human neutrophils were treated with various doses of PD 169316 and SB 525334 and assayed for chemotaxis within an fMLP gradient by EZ-TAXIScan. **Top and middle panels:** the images show the positions of cells at 0 and 12.5 min of chemotaxis; at 12.5 min, control cells approach the end of the gradient (see Figure S2B). **Bottom panels:** The images show migratory paths of individual neutrophils during an entire (30 min) chemotaxis assay. Cells are depicted in migratory tracts as circles, progressing from red to blue during increasing times of the assay. For clarity, only cells that moved in ten consecutive frames are shown. Non-moving (*e*.*g*. stationary) features are ignored by the algorithm, which compares objects among multiple images. Data are representative of three independent experiments. (See Supplementary Movie 3). (**B**) Chemotaxis parameters were calculated using data obtained from (**A**). The chemotaxis velocity was computed as the total chemotaxis path length divided by time. The chemotaxis index was computed as the ratio of net path length toward fMLP compared to the total path length.

To confirm the chemotactic inhibitory properties of both PD 169316 and SB 525334, we examined direct effects on chemotaxis of *Dictyostelium* and human neutrophils in specific linear chemoattractant gradients. *Dictyostelium* were applied to an EZ-TAXIScan channel, in a 0–0.1 μM gradient of cAMP at room temperature, in the presence of varying doses of PD 169316 or SB 525334 (Fig. 4C, Figure S2A, and Supplementary Movie 2). A similar assay of PD 169316 and SB 525334 was performed using fresh human neutrophils, under a 0–0.1 μM gradient of fMLP at 37 °C (Fig. 5A, Figure S2B, and Supplementary Movie 3). *Dictyostelium* and neutrophils rapidly polarized and migrated efficiently in control chambers in the presence of appropriate chemotactic agents. PD 169316 and SB 525334 inhibited chemotaxis velocity and directional index of both cells in dose-dependent manners, albeit with different potencies (Figs 4C,D and 5A,B). Notably, the IC_50_ values of PD 169316 and SB 525334 for *Dictyostelium* chemotaxis were similar to that determined in the original *Dictyostelium* assay screen (Fig. 4). In neutrophils, chemotaxis inhibition by PD 169316 was relatively more potent than by SB 525334 (Fig. 5B).

To assess effects of PD 169316 and SB 525334 on *Dictyostelium* chemotaxis in the absence of development, we monitored response of growing cells to folate using EZ-TAXIScan. Growing *Dictyostelium* are responsive to folate, whereas both PD 169316 and SB 525334 at 50 μM significantly impair chemotaxis (Figure S3). Stimulation of growing *Dictyostelium* with either folate or cAMP leads to the rapid, but transient, activation of mTORC2 and consequent phosphorylation of PKBR1. These responses were not impaired by either PD 169316 or SB 525334 (Figure S4). Thus, these compounds inhibit the chemotaxis of *Dictyostelium* and not simply a developmental response to cAMP.

Although compounds SB 525334 and PD 169316 are used to preferentially target (<100 nM) TGFβ receptors and p38 MAPK^43^,^44^, respectively, their specificities are not absolute^43^,^45^. Furthermore, *Dictyostelium* does not appear to have TGFβ-signaling, and although there are two ERKs in *Dictyostelium*, their normalized activations by folate or cAMP are not significantly (+/−10%) affected by either SB 525334 or PD 169316 (Figure S5).

### PD 169316 and SB 525334 inhibit human cancer cells in scratch migration assays

**Figure 6:**
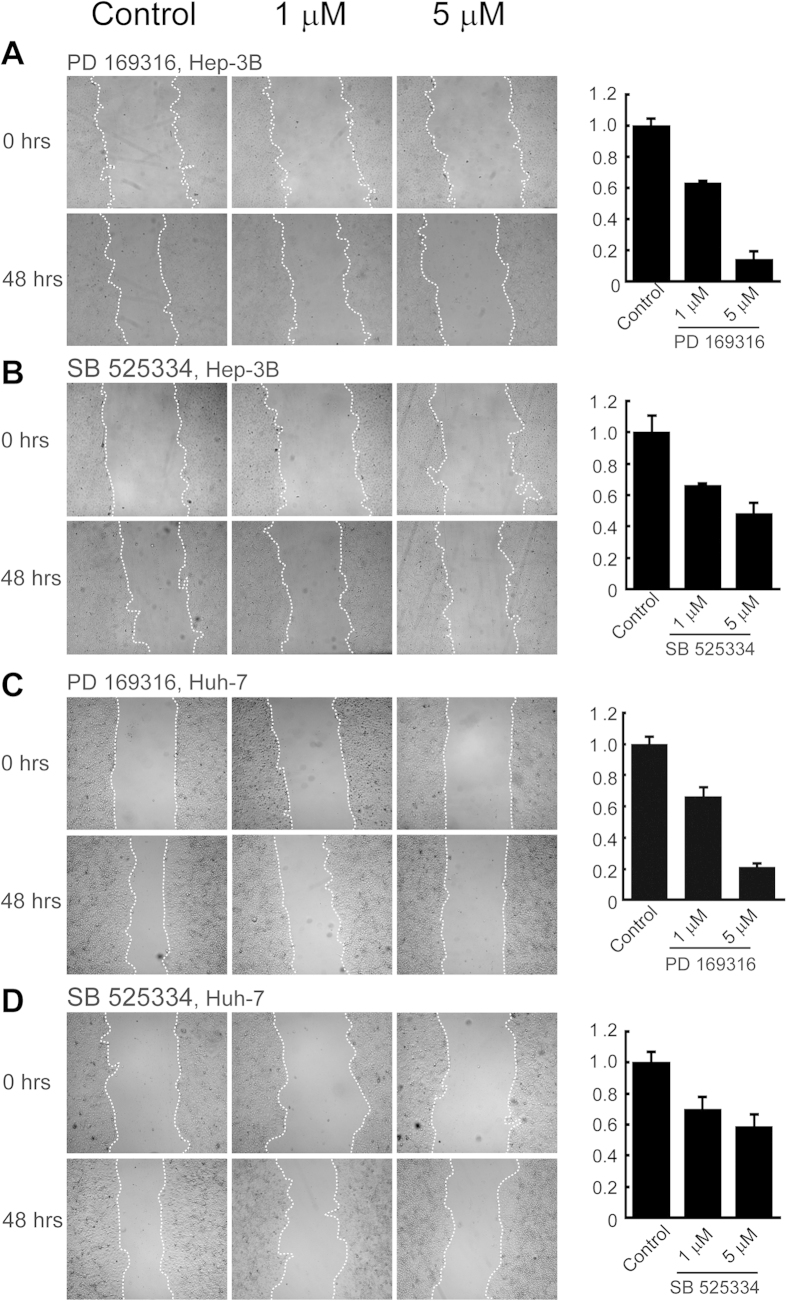
Validation of inhibitory effects of compounds PD 169316 and SB 525334 on migration of human cells in monolayer scratch assays. Hep 3B2.1–7 (**A,B**) and HuH-7 (**C,D**) hepatoma cells were grown to ~95% confluency in monolayer and straight line scratches were made across each monolayer (19). Cells were incubated with 0 (control), 1, and 5 μM PD 169316 (**A,C**) or SB 525334 (**B,D**). The exact scratch areas were recorded immediately and after two days. Invaded areas were quantified by ImageJ software. 8 Scratches were assayed in each monolayer and experiments were performed in duplicate. Error bars represent standard deviations.

To additionally assess the validity of the compounds identified, and, thus, the application of our method, we assayed the effects of PD 169316 and SB 525334 on human cancer cells during migration in monolayer scratch assays^33^. Hep 3B2.1–7 and HuH-7 Hepatoma cells were cultured in monolayer to ~95% confluence and treated with mitomycin C to arrest growth. Scratches were introduced into the cell monolayers, floating cells were removed, and fresh media was added with or without various doses of PD 169316 or SB 525334. Cell migration into scratch areas was recorded and quantified. Both PD 169316 and SB 525334 inhibited cell migration in dose dependent manners (Fig. 6). As with chemotaxis inhibition of neutrophils (see Fig. 5), cells migration in these scratch assays was more sensitive to inhibition by PD 169316 than by SB 525334.

We conclude that the HTS *Dictyostelium* assay is able to identify compounds that similarly inhibit chemotaxis/migration of human cells.

## Discussion

We have developed and validated a chemotaxis-dependent aggregation assay using *Dictyostelium* as a model chemotactic system that has capability for HTS. The phenotypic assay approach to identify small molecule inhibitors of chemotaxis in large complex libraries has unique advantages in comparison to other chemotactic assays^9^,^10^,^11^,^12^,^13^,^14^,^15^,^16^,^17^,^18^,^19^,^20^. The use of a laser scanning cytometer plate reader enabled a miniaturized assay format in 1536-well plates for the rapid detection of GFP-aggregates that quantifies chemotactic success. The assay provides a simple phenotypic approach to identify chemotaxis inhibitors of complex chemotactic processes.

Since there are large variations in the size and number of GFP aggregates per well, the 27% coefficient of variation (CV) for GFP expression may seem high. However, this is not a confounding issue. The detection threshold of the laser scanning plate cytometer is set to only capture GFP fluorescence signals from multicellular structures; the background fluorescence intensity in non-aggregated cells is zero (Figs 1 and 2A). This null background signal contrasts sharply with the several thousand GFP fluorescence units detected from the GFP aggregates formed after chemotaxis. Thus, results were in a unique format that precludes the calculation of conventional Z’ factors or signal-to-basal ratios, which approach infinity. Potent inhibitory compounds that fully suppress GFP expression/aggregate formation are readily identified from a primary screen, even though signal-to-basal ratios cannot be calculated by conventional means. In addition, for every LOPAC screening plate, we include DMSO, as negative control wells, and concentration ranges of latrunculin A, as positive inhibitory control wells. When the hundreds of these wells are classified in a binary fashion, with zero (no GFP expression) values as positive and non-zero (GFP expression) values as negative, there was perfect correlation between inhibitory and non-inhibitory effects, emphasizing the robustness of the assay.

It is interesting that partial inhibition of the GFP signal was not routinely observed. To assess this further, we monitored aggregation by direct microscopic examination of the mini-wells. The data were similar; cells either completed aggregation and expressed GFP or remained as individual cells and did not express GFP. This is not unlike the effects of cell density on the aggregation of WT *Dictyostelium*, where a two-fold cell dilution in monolayer can separate robust chemotaxis and aggregation from non-responsiveness (see Fig. 2C). These observations can be partly explained by the characteristics of *Dictyostelium* signal-response. During WT aggregation, cells must both release and respond to secreted cAMP, in a positive feedback loop^1^,^22^,^23^,^24^,^26^,^40^. There appears a density threshold, below which cells are unable to relay the signal-response and aggregation cannot proceed^46^.

The non-linearity of the assay is reflected in the latrunculin A data (see Fig. 3A). Similarly, while PD 169316 and SB 525334 inhibit *Dictyostelium* chemotaxis velocity and directionality (see Fig. 4D) in a dose-dependent manner, partial inhibition of GFP expression is often not observed. While both PD 169316 and SB 525334 were very strongly inhibitory in the screening assay, with IC_50_ values similar to that observed in the EZ-TAXIScan assay, we did not anticipate that they would inhibit chemotaxis to 100% in the directed assays. There are at least 6 intracellular signaling pathway arms that regulate chemotaxis in *Dictyostelium*^1^,^22^,^23^,^24^,^26^,^40^ and although they cross-interact, genetic ablation of several in an individual cell does not completely suppress chemotaxis^23^,^47^. Indeed, partial chemotaxis of both *Dictyostelium* and neutrophils in EZ-TAXIScan assays is observed at the highest compound concentrations tested (Figs 4C,D and 5).

Although there is a significant false positive rate due to the large number of cytotoxic compounds (*e*.*g*. 20 out of 22 primary hits), these false positives are readily recognized and eliminated using a parallel counter-screen with a highly sensitive ATP content assay for cell viability. Other false positives (*e*.*g*. inhibitors of cAMP synthesis, starvation sensing, GFP) are easily screened in direct EZ-TAXIScan or other chemotaxis assays. Whereas, not every compound identified in our initial screen is expected to be equally effective on mammalian chemotaxis, our results indicate that both of the active compounds identified from the *Dictyostelium* aggregation assay also inhibited chemotaxis/migration of human neutrophils and cancer cells. Indeed, *Dictyostelium* and mammalian cells share many pathways required for chemoattractant/chemokine responses (*e*.*g*. GPCRs, G proteins, ERKs, PKA, PI3K, TORC2, AKT, PLA_2_, PDK1, cyclases)^1^,^22^,^23^,^24^,^26^,^40^, although we recognize that not all compounds that can inhibit chemotaxis will be detected in our screen. LY-294002 may be considered such a false negative. LY-294002 is in the LOPAC library, can inhibit chemotaxis, but was not selected in our screen. At the highest concentration tested (~40 μM), LY-294002 was only partially (<50%) inhibitory in our system, consistent with LY-294002 concentrations of >100 μM required to inhibit all chemotaxtic functions (*e*.*g*. mTORC2)^35^.

The described *Dictyostelium* assay is, nonetheless, a reliable model system to identify active compounds that regulate chemotaxis, in general, and that can be further confirmed and followed in experiments with mammalian cells. *Dictyostelium* strains with specific genetic deficiencies in various chemotactic components are available and represent useful tools to dissect the mechanism of action and protein targets for the inhibitors of chemotaxis found in the phenotypic screens^1^,^22^,^23^,^24^,^48^,^49^.

In addition, this assay may also be applicable to screen for enhancers of chemotaxis. We observed that dozens of LOPAC compounds increased the number of aggregates per well (up to 18 aggregates; unpublished observations). Compounds that induce aggregation and GFP expression, under conditions normally non-permissive for *Dictyostelium* chemotaxis and aggregation [*e*.*g*. <2,000 cells/well (see Fig. 2C)] may be excellent candidates.

## Conclusions

We have developed and optimized a novel assay for high-throughput screening to identify novel inhibitors of chemotaxis. The assay uses a rapid laser-scanning cytometry plate reader for the detection of GFP-aggregates formed during *Dictyostelium* chemotaxis and a parallel cell viability counter-screen to eliminate cytotoxic compounds. The assay allowed the HTS of 1200 compounds, at 7 concentrations + controls, in ~50,000 wells with 32 (1536-well) plates. We further demonstrated that the 2 compounds identified as inhibitors of *Dictyostelium* chemotaxis are also capable of inhibiting chemotaxis/migration of human neutrophils and cancer cells. This new compound screening approach, using a phenotypic *Dictyostelium* chemotaxis-dependent aggregation assay, enables the rapid identification of lead compounds that inhibit chemotaxis in human and other cells for drug development and research tools.

## Methods

### Materials

*Dictyostelium* strain DBS0236466 (AX4 cells expressing GFP under the control of the prespore *cotB* promoter) was kindly provided by Dr. William Loomis (UCSD, La Jolla, CA)^31^. Hepatoma cell lines, Hep 3B2.1–7 and HuH-7, were purchased from Cell Bank, Chinese Academy of Sciences. One liter of D3-T media for *Dictyostelium* growth culture was composed of 7.15 g of peptone (BD Bioscience, #211693), 7.15 g of thiotone (BD Bioscience, #211921), 7.15 g of yeast extract (BD Bioscience, #212750), 15.4 g of D-glucose, 0.48 g of KH_2_PO_4_ and 0.525 g of Na_2_HPO_4_•7H_2_O. DB starvation buffer at pH 6.5 was composed of 7.4 mM NaH_2_PO_4_-H_2_O, 4 mM Na_2_HPO_4_•7H_2_O, 2 mM MgCl_2_ and 0.2 mM CaCl_2_. The ATPlite assay kit for measuring ATP content in cells as a cytotoxicity indicator was purchased from Perkin Elmer (#6016731). Latrunculin A was purchased from Sigma-Aldrich (#L5163). Hygromycin B and the Library of Pharmacologically Active Compounds (LOPAC) containing 1280 compounds were also purchased from Sigma-Aldrich. MEM media were composed of MEM (GIBCO, #41500034), 1.5 g/L of NaHCO_3_, 0.11 g/L of Sodium Pyruvate and 10% of fetal bovine serum (GIBCO, #10437-028). DMEM media was composed of DMEM (GIBCO, #12800017), 1.5 g/L of NaHCO_3_ and 10% of fetal bovine serum.

### Cell Growth and Starvation

*Dictyostelium cotB*/*GFP* cells^31^ were grown to confluence in nutrient abundant D3-T media in stationary flasks at 20 °C. Cells were diluted into fresh D3-T media at 2 × 10^5 ^cells/ml and cultured in shaking flasks for 2 days at 220 rpm, 20 °C). Cells at log-phase (1–3 × 10^6 ^cells/ml) were harvested by centrifugation at 1000 × g for 5 min, washed twice with DB, and resuspended in DB at appropriate cell densities.

Hepatoma cell lines, Hep 3B2.1–7 and HuH-7, were cultured with MEM and DMEM media in a humidified 5% CO_2_-95% air atmosphere incubator at 37 °C.

### Preparation of Compound Plates

All of the compounds were dissolved in 100% DMSO at 10 mM stock solutions. For the LOPAC library, the compounds were serially diluted with DMSO in 384-well plates at a 1:5 titration ratio over 7 concentrations and then formatted to 1536-well compound plates using an Evolution P3 system (PerkinElmer, Wellesley, MA). The stock solutions of control compounds and the selected compounds were diluted with DMSO in 384-well plates at a 1:3 titration ratio over 12 concentrations, and then formatted to 1536-well compound plates^50^. The final concentrations of each compound ranged from 2.46 nM to 38.3 μM in the chemotaxis-dependent aggregation assay (0.38% DMSO in all wells) and from 3.68 nM to 57.5 μM in the ATP content assay (0.58% DMSO in all wells).

### Data Analyses

Screening data were normalized and concentration-effect relationships derived using NCGC-developed software^51^. IC_50_ values were obtained by data fit using a residual error minimization algorithm with automatic outlier determination to a 4-parameter Hill. Concentration-effect relationships (CERs) were categorized by fit quality (r^2^), response magnitude, and degree of measured activity^51^. For validation, data were analyzed using GraphPad Prism software. Percent inhibition was derived from normalization of the Logarithms of positive and negative controls within the same experimental set. IC_50_ values were obtained from nonlinear curve fits using a sigmoidal dose−response model.

### EZ-TAXIScan Chemotaxis Assay

The EZ-TAXIScan chemotaxis assays are modifications^32^. For *Dictyostelium* chemotaxis, log phase cells (1–3 × 10^6 ^cells/ml) were harvested, washed twice and resuspended in DB starvation buffer at 2 × 10^7 ^cells/ml. Cells were shaken at 125 rpm at 20 °C and pulsed with 75 nM final concentration of cAMP every 6 min. After one hour of cAMP pulsing, various doses of SB 525334, PD 169316, or an equivalent amount of DMSO as a control was added to the cells and pulsing was continued for additional four and half hours. Cells were then applied onto the EZ-TAXIScan (Effector Cell Institute, Tokyo, Japan) channel with 0–0.1 μM gradient of cAMP in the chemoattractant well at room temperature.

Blood samples were obtained from paid healthy volunteers who gave written informed consent to participate in an IRB-approved study for the collection of blood samples for *in vitro* research use. The protocol is designed to protect subjects from research risks as defined in 45CFR46 and to abide by all NIH guidelines for human subjects research (protocol number 99-CC-0168). Neutrophils were isolated from heparinized human whole blood by dextran sedimentation and differential centrifugation over Histopaque 1077 (Sigma-Aldrich) as described previously^32^. Residual erythrocytes were removed by hypotonic lysis. The fresh-isolated neutrophils were treated with PD 169316, SB 525334, or an equivalent amount of DMSO as a control was added to the cells for 4 hrs. Cells were then applied onto the EZ-TAXIScan channel with 0–0.1 μM gradient of fMLP in the chemoattractant well at 37 °C.

Migratory *Dictyostelium* or neutrophil cells were recorded every 15 sec for 30 min^32^. Cell velocity and chemotaxis index were calculated with MATLAB software, as described^32^.

For *Dictyostelium* chemotaxis to folate, log phase cells (1–3 × 10^6 ^cells/ml) were incubated for 30 min. with 50 μM SB 525334, 50 μM PD 169316, or an equivalent amount of DMSO (0.16%) as a control. Cells were then applied onto the EZ-TAXIScan channel with 0–100 μM gradient of folate in the chemoattractant well at room temperature.

### Monolayer Scratch Migration Assay

The assay is modified from a previous report^33^. Hep 3B2.1–7 and HuH-7 Hepatoma cells were grown in monolayer, 6-well plates to ~95% confluency and treated with mitomycin C to arrest cell growth. Two straight scratches were made with a pipette tip across each cell monolayer. Floating cells were removed by washing with PBS and fresh media, with varying doses of PD 169316, SB 525334, or an equivalent amount of DMSO, were added into each well. 8 areas of scratches lines were marked for each cell monolayer and immediately recorded by camera; after two days, the exact scratch areas were again recorded. The original and resulting areas absent of cells were quantified by ImageJ software. The 8 areas of each well were averaged and variation further assessed with duplicate wells for each treatment. Normalization was relative to % control invasion, set to 1.0.

### Phosphorylation assays

cAMP and folate stimulations of PKBR1 and ERK phosphorylation were assayed by immunoblot, as previously described^52^,^53^.

### Trypan blue staining

Control and latrunculin A treated cells were incubated with 0.1% trypan blue (Lonza) for 10 min at room temperature^39^ and cell viability quantified using a Nexcelom Cellometer and light microscopy.

## Additional Information

**How to cite this article**: Liao, X.-H. *et al.* A High-Throughput, Multi-Cell Phenotype Assay for the Identification of Novel Inhibitors of Chemotaxis/Migration. *Sci. Rep.* **6**, 22273; doi: 10.1038/srep22273 (2016).

## Supplementary Material

Supplementary Information

Supplementary Movie 1

Supplementary Movie 2

Supplementary Movie 3
