# Supplementary material for: A High-Throughput, Multi-Cell Phenotype Assay for the Identification of Novel Inhibitors of Chemotaxis/Migration
Source: Sci Rep. 2016 Mar 9;6:22273. doi: 10.1038/srep22273 (PMC4783656; doi:10.1038/srep22273)
Supplement: Supplementary Information [file srep22273-s1.pdf]

A High-Throughput, Multi-Cell Phenotype Assay for the Identification of Novel Inhibitors of Chemotaxis/Migration

Xin-Hua Liao[1,2], Netra Pal Meena[2], Noel Southall[3], Lunhua Liu[4], Manju Swaroop[3], Arina Li Zhang[1], Jan Jian Xiang[1], Carole A. Parent[4], Wei Zheng[3], and Alan R. Kimmel[2]*

[1] Institute for Translational Medicine, School of Basic Medical Sciences, Fujian Medical University, Fuzhou, Fujian 350108, China

[2] Laboratory of Cellular and Developmental Biology, National Institute of Diabetes and Digestive and Kidney Diseases

[3] Therapeutics for Rare and Neglected Diseases, National Center for Advancing Translational Sciences

[4] Laboratory of Cellular and Molecular Biology, National Cancer Institute

[2,3,4] The National Institutes of Health, Bethesda, MD 20892, USA

* Telephone: (301) 496-3016
Facsimile: (301) 496-5239
e-mail: alank@helix.nih.gov

Table of Contents

- Supplementary Tables S1-S2
- Supplementary Figures S1-S5
- Supplementary Movie Legends

SUPPLEMENTARY TABLES

Supplementary table 1

Protocol for *Dictyostelium* chemotaxis/aggregation assay in 1536-well plates

Step	Parameter	Value	Description
1	Reagent - 1	3 μL	DB starvation buffer
2	Compound	23 nL	Compound library or control plate
3	Reagent - 2	3 μL	Cells in DB buffer at 2.67×10^6 cells/ml (8,000 cells/well)
4	Incubation	48 hours	Room temperature (20-23.5 °C)
5	Detection	Ex=455-488 nm<br>Em=500-530 nm	Acumen ^{e}X3; 10 min/plate

Supplementary table 2

Protocol for *Dictyostelium* ATP content assay in 1536-well plates

Step	Parameter	Value	Description
1	Reagent - 1	2 μL	DB starvation buffer
2	Compound	23 nL	Compound library or control plate
3	Reagent - 2	2 μL	Cells in DB buffer at 2.5×10^5 cells/ml (500 cells/well)
4	Incubation - 1	72 hours	Room temperature (20-23.5 ° C)
5	Reagent -3	3 μL	ATPlite reagent
6	Incubation -2	5 minutes	Shake at 500 rpm
7	Detection	luminescence	Viewlux; 1 min/plate

SUPPLEMENTARY FIGURES

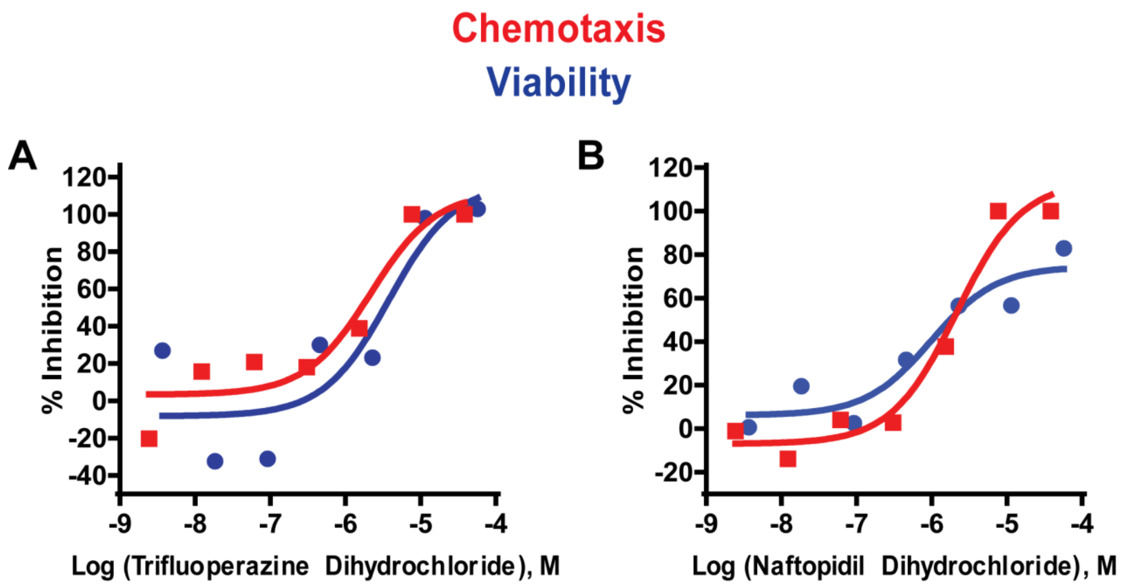

Figure S1. Cytotoxic compounds identified from LOPAC test screen using *Dictyostelium* chemotaxis-dependent aggregation and ATP content assays.

A and B. Concentration response of two representative compounds from a LOPAC screen, Trifluoperazine Dihydrochloride **(A)** and Naftopidil Dihydrochloride **(B)**, with similar inhibitory effects on chemotaxis/aggregation (via GFP) and viability (via ATP).

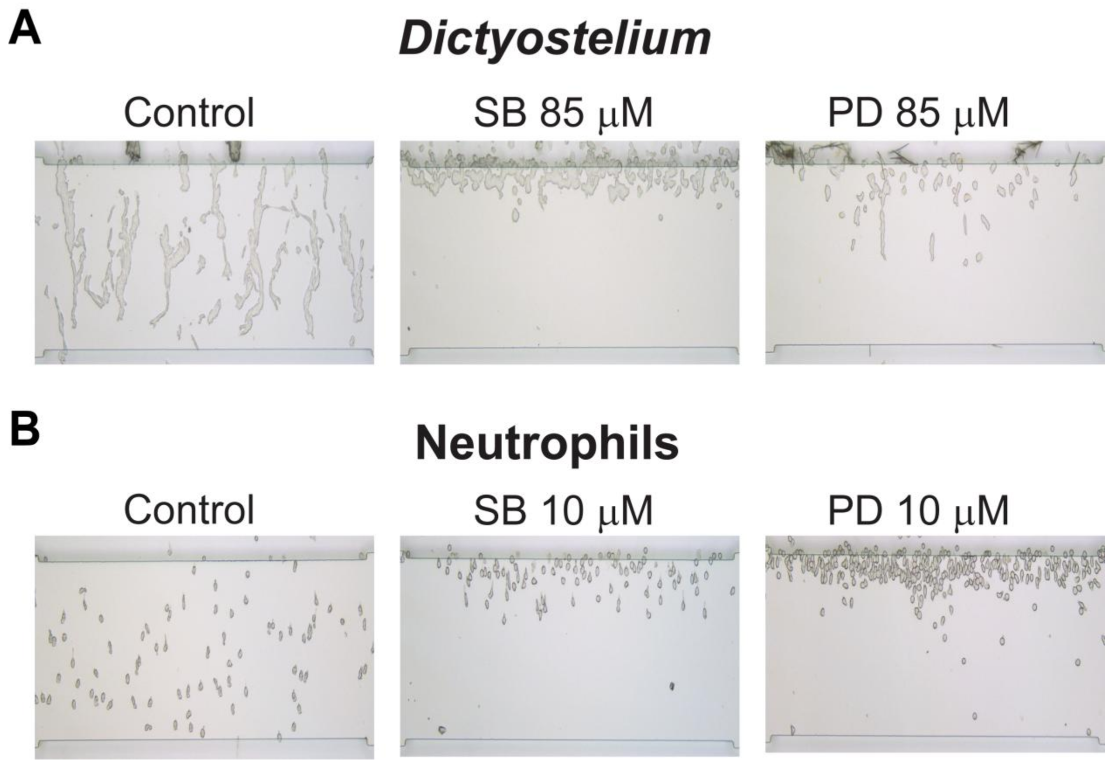

Figure S2. Validation of inhibitory effects of compounds PD 169316 and SB 525334 on *Dictyostelium* and neutrophil chemotaxis in EZ-TAXIScan assays.

Expanded images of **(A)** *Dictyostelium* (from Figure 4C) and **(B)** neutrophils (from Figure 5A), after 25 and 12.5 min. migration, respectively.

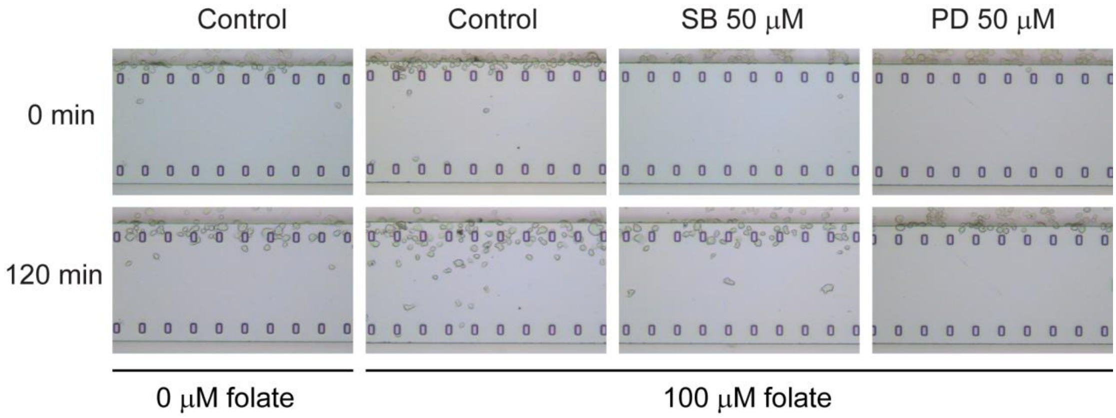

Figure S3. Validation of inhibitory effects of compounds PD 169316 and SB 525334 on the chemotaxis of growing *Dictyostelium* to folate.

Growing *Dictyostelium* were pre-treated with or without 50 μM PD 169316 or SB 525334 for 30 min and assayed for chemotaxis within a folate gradient by EZ-TAXIScan. Top and middle panels show images of cells at 0 and 2 hrs of chemotaxis. Control cells do not migrate directionally in absence of a folate gradient.

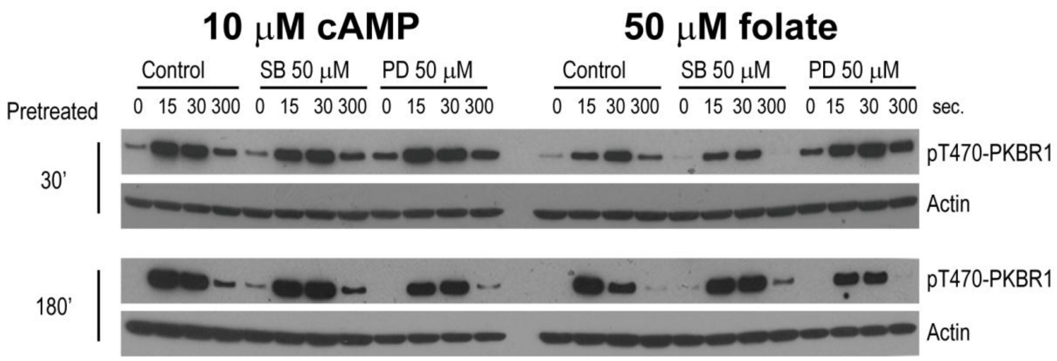

Figure S4. mTORC2 phosphorylation of PKBR1 in *Dictyostelium* is not inhibited by compounds PD 169316 and SB 525334.

Growing *Dictyostelium* were pre-treated with or without 50 μM PD 169316 or SB 525334 for 30 or 180 min and then stimulated with saturating doses of cAMP or folate. mTORC2 phosphorylation of PKBR1 was assayed by immunoblotting, at the times indicated.

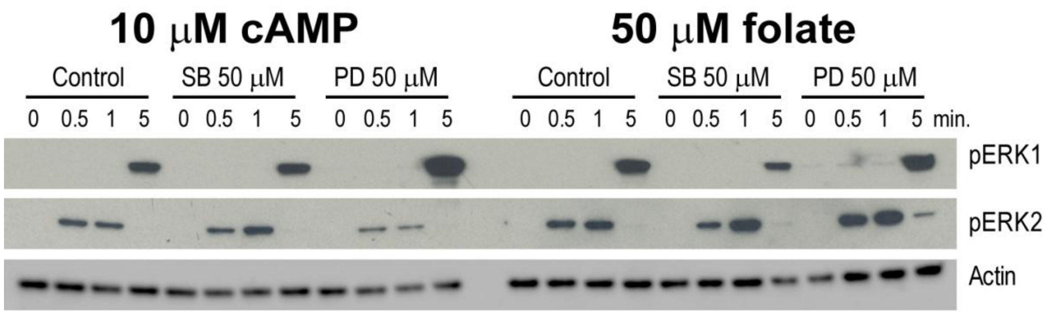

Figure S5. ERK1 and ERK2 phosphorylations in *Dictyostelium* are not inhibited by compounds PD 169316 and SB 525334.

Growing *Dictyostelium* were pre-treated with or without 50 μM PD 169316 or SB 525334 and then stimulated with saturating doses of cAMP or folate. ERK1 and ERK2 phosphorylation was assayed by immunoblotting, at the times indicated.

SUPPLEMENTARY MOVIE LEGENDS

Movie 1. Time course expression of the multicellular reporter GFP during chemotaxis and aggregation of *Dictyostelium*.

Cells were incubated in starvation buffer for various times and visualized by differential interference contrast (DIC) and fluorescent confocal (GFP) microscopy (see Figures 1A and 2A). Left Panel: GFP; Middle Panel: DIC; Right Panel: merged.

Movie 2. Chemotaxis of *Dictyostelium* in the presence of various concentrations of SB 525334 or PD 169316 under a cAMP gradient using EZ-TAXIScan.

Migration of *Dictyostelium* during an entire (30 min) chemotaxis assay in the absence or presence of SB 525334 (5 μM, 20 μM, 85 μM) or PD 169316 (8 μM, 85 μM), from top to bottom (see Figure 5C).

Movie 3. Chemotaxis of neutrophils in the presence of various concentrations of PD 169316 or SB 525334 under an fMLP gradient using EZ-TAXIScan.

Migration of neutrophils during an entire (30 min) chemotaxis assay in the absence or presence of PD 169316 (1 μM, 5 μM, 10 μM) or SB 525334 (5 μM, 10 μM), from top to bottom (see Figure 6A).
